# Flow Cytometric Determination of Actin Polymerization in Peripheral Blood Leukocytes Effectively Discriminate Patients With Homozygous Mutation in ARPC1B From Asymptomatic Carriers and Normal Controls

**DOI:** 10.3389/fimmu.2019.01632

**Published:** 2019-07-16

**Authors:** Andreja N. Kopitar, Gašper Markelj, Miha Oražem, Štefan Blazina, Tadej Avčin, Alojz Ihan, Maruša Debeljak

**Affiliations:** ^1^Faculty of Medicine, Institute of Microbiology and Immunology, University of Ljubljana, Ljubljana, Slovenia; ^2^Department of Allergology, Rheumatology and Clinical Immunology, University Children's Hospital, University Medical Center Ljubljana, Ljubljana, Slovenia; ^3^Department of Radiation Oncology, Institute of Oncology Ljubljana, Ljubljana, Slovenia; ^4^Department of Pediatrics, Faculty of Medicine, University of Ljubljana, Ljubljana, Slovenia; ^5^Unit for Special Laboratory Diagnostics, University Children's Hospital, University Medical Center Ljubljana, Ljubljana, Slovenia

**Keywords:** ARPC1B deficiency, Arp2/3, actin polymerization, flow cytometry, functional test, peripheral blood leukocytes

## Abstract

Actin nucleators initiate formation of actin filaments. Among them, the Arp2/3 complex has the ability to form branched actin networks. This complex is regulated by members of the Wiscott-Aldrich syndrome protein (WASp) family. Polymerization of actin filaments can be evaluated through flow cytometry by fluorescent phalloidin staining before and after stimulation with N-formyl-methionyl-leucyl-phenylalanine (fMLP). We identified a missense mutation in the gene ARPC1B (Arp2/3 activator subunit) resulting in defective actin polymerization in four patients (three of them were related). All patients (1 male, 3 female) developed microthrombocytopenia, cellular immune deficiency, eczema, various autoimmune manifestations, recurrent skin abscesses and elevated IgE antibodies. Besides four patients with homozygous mutation in ARPC1B, we also identified six heterozygous carriers without clinical disease (3 males, 3 females) within the same family. We developed a functional test to evaluate Arp2/3 complex function, which consists of flow cytometric detection of intracellular polymerized actin after *in vitro* fMLP stimulation of leukocytes. Median fluorescence intensities of FITC-phalloidin stained actin were measured in monocytes, neutrophils and lymphocytes of patients, carriers, and healthy control subjects. We detected non-efficient actin polymerization in monocytes and neutrophils of homozygous patients compared to carriers or the healthy subjects. In monocytes, the increase in median fluorescence intensities was significantly lower in patients compared to carriers (104 vs. 213%; *p* < 0.01) and healthy controls (104 vs. 289%; *p* < 0.01). Similarly, the increase in median fluorescence intensities in neutrophils was significantly increased in the group with carriers (208%; *p* < 0.01) and healthy controls (238%; *p* < 0.01) and significantly decreased in the patient's group (94%). Our functional fMLP/phalloidin test can therefore be used as a practical tool to separate symptomatic patients from asymptomatic mutation associated to actin polymerization.

## Introduction

Polymerization of actin plays an important role in many immune functions like proliferation and differentiation of immune cells, migration, intercellular and intracellular signaling and activation of both, innate and adaptive immune responses. Dynamic rearrangement of cell shape relies on rapid assembly and disassembly of filamentous actin (1). To initiate actin assembly during such processes, cells generate free barbed ends that act as templates for polymerization by uncapping or severing existing filaments or by nucleating from monomers *de novo* (2). The microfilamentous cytoskeleton is a highly dynamic network that is made of actin and numerous actin-associated proteins (3). Polymerization is initiated by three classes of actin nucleators, the actin related protein 2/actin related protein 3 (Arp2/3) complex, the formin family, and the more recently identified Spire, cordon-bleu, and leiomodin family (1). They promote nucleation, in response to specific upstream signals such as integrin activation, T-cell and B-cell receptor ligation and chemokine stimulation (4). Each class of nucleators has a distinct mechanism for initiating actin polymerization (1). The first major actin nucleator to be discovered was the Arp2/3 complex, which is composed of evolutionarily-conserved subunits including the actin-related proteins Arp2 and Arp3 and five additional subunits ARPC1–5 (5). Arp2/3 complex is a macromolecular machine that nucleates branched actin filaments in response to cellular signals. Wiskott-Aldrich syndrome proteins (WASp) family regulates the nucleation activity of Arp2/3 complex, providing a way for cells to assemble branched actin filament networks (6). Rho-family GTPases like Cdc42 and Rac2 are involved in regulation of actin polymerization by directly interacting with WASp (7).

Control of actin dynamics is essential to many cellular processes, including motility, vesicle trafficking, and cell division. The Arp2/3 complex nucleates new (daughter) filaments on the sides of existing (mother) filaments in response to activating stimulus from the WASp family [reviewed in Smith et al. (8)]. Dynamic associations of Arp2/3 complex with mother filament and WASp is temporally coordinated to initiation daughter filament growth which is necessary to perform a variety of cellular functions including motility (9). Phalloidin has been widely used for studying actin polymerization in biochemical assays and in fluorescent microscopy (10). It is a small toxic molecule produced by the poisonous mushrooms *Amanita phalloides*. Phalloidin stabilizes actin structures and therefore prevents the depolymerization of the actin polymers, resulting in cytotoxicity (11).

Over 300 genes have been causally linked to monogenic forms of primary immunodeficiency disorder (PID), including a number that are associated with actin polymerization are mostly due to genetic defects in such regulatory proteins. Known cytoskeleton-associated PIDs present either as combined or severe combined immunodeficiencies or as phagocyte disorders (4).

Our study focused on ARPC1B deficiency, a recently described PID (12, 13). Disease is characterized by recurrent infections, hypersensitivity, autoimmunity and increased risk of malignancies. Majority of the patients have increased bleeding tendency due to thrombocytopenia and platelet disfunction. Recurrent infections are both bacterial and viral most commonly in the respiratory tract, skin and gastrointestinal tract. Hypersensitivity features include eczema, food allergies, and asthma. Predominant autoimmune features are skin vasculitis and inflammatory bowel disease. Disease clinically resembles with loss-of-function mutations in WAS gene (13, 14).

The aim of our study was to introduce a rapid, very specific and simple functional test to evaluate impaired actin polymerization in patients with mutation in ARPC1B. Intracellularly polymerized actin was measured by flow cytometry with fluorescent phalloidin before and after *in vitro* fMLP stimulation of leukocytes.

## Methods

### Patients/Study Design

In the period from November 2016 to December 2018, we evaluated patients that were initially identified as having Wiskott-Aldrich like syndrome (thrombocytopenia, eczema, variable degree of immunodeficiency), but later ARPC1 mutation were identified. Four patients with homozygous ARPC1B mutation (1 male, 3 females), 6 heterozygous carriers without clinical disease (3 males, 3 females) within the same family and twelve healthy subjects (without ARPC1B mutation) were included in the study. Clinical and immunological parameters of the patients are summarized in Tables 1, 2.

**Table 1:** Clinical parameters of the patients with ARPC1B mutation at the first evaluation.

**Patient**	**Age**	**Infections**	**Allergy**	**Autoimmunity**	**Malignancy**	**Other**
P1	27 year	Recurrent bonchiolitis and pneumonias, recurrent skin abscesses	eczema, Food, pollen and mites allergy	Enterocolitis, Small vessels vasculitis, Autoimmune thrombocytopenia, panniculitis	/	Stunted growth
P2	24 year	Prolonged pneumonias, Gastroenteritis, Candida esophagitis, Chronic warts - Epidermodysplasia verruciformis	Eczema, Food allergy	Enterocolitis, Pernicous anemia	Metaplasia in gastric mucosa, bowel adenoma *in situ*	Stunted growth
P3	30 year	Recurrent pneumonias, lung abscesses, Gastroenteritis, Recurrent skin abscess, chronic leg ulceration, Gastroenteritis, Genital condyloma and severe warts	Eczema, Food, mites, animal epithelia allergy, Allergic asthma	Enterocolitis, Small vessels vasculitis	Cervical intraepithelial neoplasia grade 2	Stunted growth
P4	16 months	/	Mild eczema	Evans syndrome	/	/

**Table 2:** Immunological parameters of the patients with mutations in ARPC1B at the first evaluation.

**Patient**	**IgE**	**IgG**	**IgA**	**IgM**	**CD3**	**CD19**	**CD4**	**CD8**	**NK**	**T cell prolif. (PHA)**	**T cell prolif. (CD3+CD28)**
	**IE/L**	**g/L**			**10**^****9****^ **cells/L**					**%**	**%**
NV1	0–100	7.0–16.0	0.7–5.0	0.4–2.8	0.7–1.9	0.1–0.4	0.4–1.3	0.2–0.7	0.04–0.2	29–57	50–85
P1	1746↑	13.4	0.98	2.52	0.626↓	0.562↑	0.305↓	0.498	0.369	15↓	52
P2	932↑	11.3	4.7	0.85	0.967	0.521↑	0.459	0.546	0.468	22↓	70
P3	716↑	13.6	6.3↑	0.6	1.006	0.542↑	0.730	0.126↓	0.066↓	33	66
NV2	0-60	4.7-12.0	0.14-0.91	0.4-1.5	2.2-5.5	0.9-2.5	1.1-3.6	0.5-1.8	0.1-1.1	29-57	50-85
P4	<19	12.4↑	2.21↑	1.33	1.551↓	2.066	1.024↓	0.408↓	0.777	38	77

*Immunoglobulin classes were measured in serum. The concentration of IgE is in IE/L, and IgG, IgA, IgM in g/L. The concentration of lymphocytes subpopulations are presented as ×10^9^ cells/L, the proliferation of T lymphocytes after stimulation with PHA or CD3 and CD28 in percentage of proliferating cells. CD3, T lymphocytes; CD19, B lymphocytes; CD4, helper T cells; CD8, cytotoxic T cells; NK, Natural killer cells; PHA, phytohaemagglutinin; anti-CD3, anti-CD28 – monoclonal antibodies, P, patients, NV1, normal values for adults, NV2, normal values for 16 month. An arrow indicates deviation from normal values (15, 16)*.

### Genetic Tests

We extracted genomic DNA from whole blood EDTA samples of four patients and their relatives (siblings, parents, grandparents) according to established laboratory protocols using FlexiGene DNA isolation kit (Qiagen, Germany). We performed whole exome sequencing of an index patient in collaboration with Eurofins Genomics (Ebersberg, Germany) using Ion AmpliSeq Exome kit for whole exome enrichment preparation and Ion PI™ Sequencing 200 Kit v3 together with Ion Proton Sequencer (Life Technologies, USA) to perform whole exon sequencing. We analyzed genetic variants with coverage >15x with Variant Studio 2.2 software (Illumina). Since pathogenic mutations leading to WAS-like are likely rare in unaffected populations, we filtered out all variants identified from the latest draft of the 1000 Genomes Project and dbSNP build 132. The search tool for the retrieval of interacting genes/proteins (STRING, http://string-db.org/) was used to construct protein-protein interactions, which are involved downstream and upstream of the WASP protein, and could be involved in defective actin reorganization, cell trafficking and synapse formation. We directed and focused the analysis on actin reorganization defects using the panel of genes (*ACTR2, ACTR3, ARPC1A, ARPC1B, ARPC2, ARPC3, ARPC4, ARPC5, ARPC5L, BTK, FYN, GRB2, NCK1, PSTPIP, WIPF1*). We excluded from further analysis all variants exceeding the threshold value for known variant minor allele frequency at 1%. We used the autosomal recessive inheritance model to further reduce the number of potential causative variants. We confirmed the identified candidate variant and its family segregation by a targeted Sanger sequencing run on ABI Genetic Analyzer 3500 (Applied Biosystems, USA) using custom oligonucleotides and BigDye Terminator v3.1 sequencing kit (Applied Biosystems, USA). We analyzed the potential deleterious effect of identified genetic variant with several *in silico* prediction tools: SIFT (Sorting Intolerant from Tolerant; http://sift.bii.a-star.edu.sg), Polyphen2 (http://genetics.bwh.harvard.edu/pph2/), CADD score (http://cadd.gs.washington.edu/score) and Mutation taster (http://www.mutationtaster.org/).

### Functional fMLP/Phalloidin Test

The actin polymerization was determined by a flow cytometric assay. Fifty microliter of citrated whole blood was incubated for 20 s with or without 10 μl of N-formyl-methionyl-leucyl-phenylalanine (fMLP) (final concentration 0.83 × 10^−3^M). The stimulation time with fMLP was selected among different incubation times; 10, 20, 30, 40, 50, 60, 120, and 180 s as optimized for cell viability and phalloidin mean fluorescence intensity (MFI). Thereafter cells were fixed with 50 μL 4% formaldehyde and incubated for 25 min at room temperature. Red blood cells were lysed with 2 mL BD FACSTM Lysing Solution (BD Biosciences, San Jose, CA, USA) for 10 min, centrifuged (5 min, 450 g) and resuspended in 100 μl PBS containing 1% BSA. Cells were then stained with 10 μL CD14 PE (cat. No. 345785; clone MφP9) and 10 μL CD45 PerCP-Cy5.5 (cat. No. 332784: clone 2D1) monoclonal antibodies (Becton Dickinson, CA, USA) and incubated in the dark for another 15 min. Cells were then permeabilized with 1 mL BD Perm/WashTM buffer (BD Biosciences), centrifuged and intracellularly stained with 5 μL of 1,5 μM FITC-phalloidin, final concentration 150 nM (Sigma-Aldrich, USA). The optimal dilution of phalloidin was determined by titration. Following 60 min incubation in dark at 4°C, the sample was washed twice with 1 mL of PBS containing 1% BSA before analysis by flow cytometry within 30 min. Samples were evaluated by a BD FACS Canto I or II cytometer (BD Biosciences, CA, USA), using DIVA software (BD Biosciences, San Diego, USA). At least 10,000 cells were acquired. The data analysis was performed using FlowJo version 10.1 software (TreeStar, Ashland, USA). The cells were gated into monocytes, neutrophils and lymphocytes according to CD45-PerCP Cy5.5 and CD14-PE distribution (Figure 2). The gates were checked by backgating on scatter plots. The FITC-phalloidin fluorescence on monocytes, neutrophils and lymphocytes was displayed on histograms. Patient samples were always measured in duplicates. Normalization was performed by setting the fluorescence intensity of unstimulated samples to 100%. Increase in MFI in the fMLP stimulated samples was calculated as $\frac{stimulated\;sample^{*}100}{unstimulated\;sample}$.

Apoptotic and viable cells we distinguished by 7-AAD vs. annexin V-APC (both from BD Pharmingen) staining. Aggregated doublet cells were excluded from analysis.

### Morphological Assay for Actin Polymerization

Mononuclear cells were isolated on a Ficoll–Hypaque gradient. Distribution of F-actin was evaluated in cells fixed with 4% formaldehyde, centrifuged on slides and air dried. After washing with PBS, cells were stained with 100 μL 5M FITC-phalloidin. Labeled samples were again washed in PBS and mounted using VECTASHIELD® mounting medium with DAPI. Slides were examined at 400-or 1,000-fold magnification on a DMRB Leica fluorescence microscope.

### Burst Test

The quantitative determination of leukocyte oxidative burst was performed in heparinized whole blood according to instructions of the manufacturer (Glycotope Biotechnology GmbH, Heidelberg, Germany). Briefly, 100 μL of whole blood were stimulated with unlabelled opsonized bacteria (*Escherichia coli*) as a particular stimulator, protein kinase C ligand phorbol 12-myristate 13-acetate (PMA) as a strong stimulus, or fMLP as low stimulant. This stimulations induce monocytes and granulocytes to produce reactive oxygen metabolites. Radical formation was measured at 37°C by conversion of dihydrorhodamine 123 to the fluorescent rhodamine 123. A sample without stimulus served as negative background control. The reaction was stopped by addition of lysing solution, which also removed erythrocytes. After a washing step, DNA staining was performed to exclude aggregation artifacts. Cells were analyzed by flow cytometry (FACSCanto; Becton Dickinson), using DIVA software (Becton Dickinson) for data acquisition and analysis, and the results were expressed as MFI plotted on histograms (17).

### Phagocytosis Test

To determinate the phagocytosis of neutrophil granulocytes and monocytes the cells were ingesting FITC-labeled opsonized *E. coli* according to instructions of the manufacturer. We used PHAGOTEST kit from Glycotope Biotechnology GmbH. Briefly, heparinized whole blood was incubated with bacteria at 37°C, a negative control sample remains on ice. The phagocytosis was stopped by placing the samples on ice and adding quenching solution, which discrimination between attachment and internalization of bacteria. Erythrocytes were lysed and DNA staining solution was added just before measurement. Cells were analyzed on FACSCanto flow cytometer, using DIVA software (Becton Dickinson). The percentage of cells, which ingested the FITC labeled *E. coli*, was determinate using the gate on negative control sample on FITC fluorescence histogram.

### Statistical Analysis

Increase in median fluorescent intensity was analyzed with independent two-tailed Student's t-test. Statistics were presented as mean of the results ± standard error of the mean (SEM). *P* < 0.05 were considered statistically significant. Whenever two or more replicate samples where measured on the same day for the same donor, we used the calculated average value of the measurements.

## Results

### Patients

All patients and carriers came from the north-east Slovenian Roma community. Three patients presented in neonatal period with eczema, thrombocytopenia and bloody diarrhea. Later they developed recurrent bacterial infections of lung and skin, therapy resistant inflammatory bowel disease, allergic reactions and other various autoimmune features (AI vasculitis, AI thrombocytopenia, and pernicious anemia). Forth patient presented with mild excema and autoimmune hemolytic anemia and thrombocytopenia (Evans syndrome) at 7 months. They had decreased IgM and elevated IgA and IgE antibodies, a mild cellular immune deficiency, slightly decreased phagocytic assays. Clinical characteristics of the patients with mutations in ARPC1B are summarized in Table 1.

### Genetic Studies

Whole exome sequencing identified a mutation in ARPC1B gene (Arp2/3 activator), resulting in defective actin polymerization.

A novel mutation fits in an autosomal recessive model of inheritance. In ARPC1B gene which codes for p41protein in ARP2/3 complex the homozygous substitution c.265A>C was found. It changes amino acid threonine at position 89 into proline (p.Thr89Pro). We predicted the mutation pathogenicity using several *in silico* programs: SIFT (deleterious 0.01), Polyphen2 (possibly_damaging 0.903), CADD score (25.5) and Mutation taster (disease causing 0.999). We cite nomenclature according to the HGVS guidelines (www.hgvs.org/mutnomen). Sequence variants were checked using the Mutalyzer program (http://www.LOVD.nl/mutalyzer). The variant was confirmed with Sanger sequencing. Variant is not present in dbSNP or ExAC database (Exome Aggregattion Consortium: http://exac.broadinstitute.org/). Another three patients were also homozygous for the c.265A>C substitution.

Family segregation analysis was performed in several family members. Parents of all patients are heterozygous carriers of the mutation and have not developed clinical signs of the disease.

**Figure 1:**
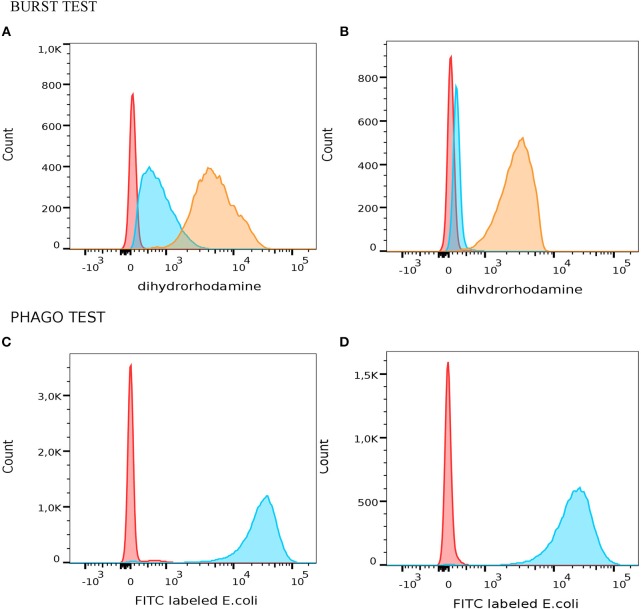
Measurement of oxidative burst and phagocitosis in granulocytes with PHAGOBURST^TM^ test (Glycotope Biotechnology GmbH, Heidelberg, Germany) by flow cytometer. A representative example of functional granulocyte activity which produced different amount of reactive oxidants in patient with mutation in ARPC1B **(A)** and healthy control **(B)**. Red – unstimulated granulocytes; blue – fMLP stimulated granulocytes; orange – PMA stimulated granulocytes. Different phagocytic activity of granulocytes from patients with mutation in ARPC1B **(C)** and healty control **(D)** after phagocytosis of *E. coli* (blue histogram) and negative control (red histogram).

All patients with mutation in ARPC1B had slightly decreased phagocytosis and normal oxidative burst after stimulation of monocytes and granulocytes with opsonized *E. coli* or PMA. However, we observed increased respiratory burst in neutrophils after mild stimulation with fMLP in average 67% (Figure 1).

### Functional fMLP/Phalloidin Test

**Figure 2:**
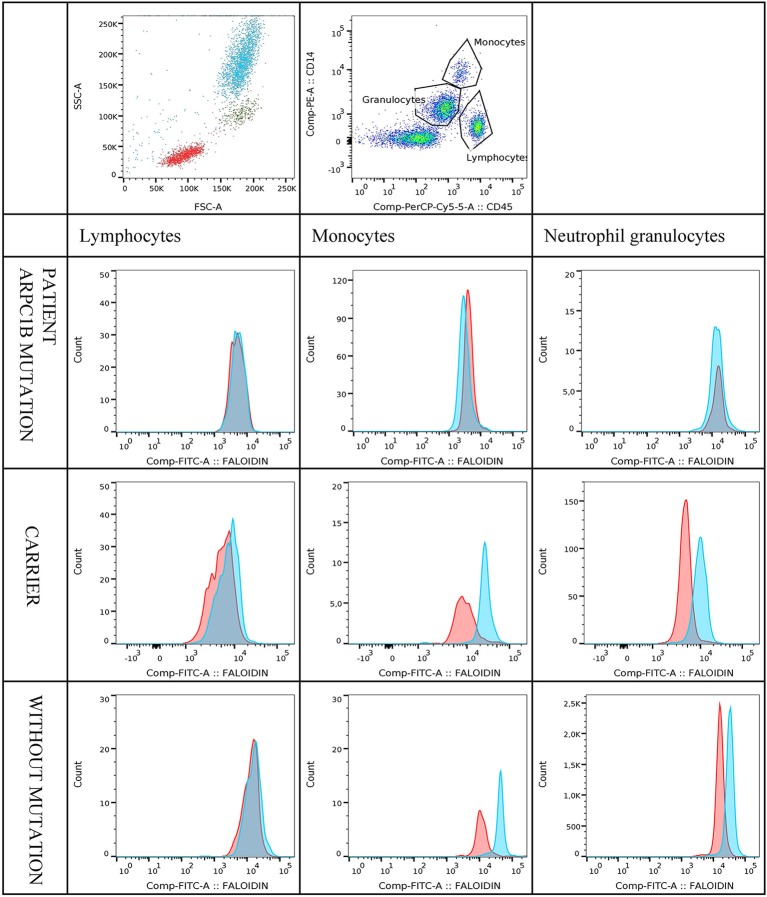
Figure shows an example of multicolour staining and flow cytometry analysis of FITC-phalloidin staining. In the representative pseudocolor plot, leukocytes are separated according to CD45 vs. CD14 staining and gated on lymphocytes, monocytes and neutrophil granulocytes. Histograms show the staining profile of intracellular actin in unstimulated (red) and fMLP stimulated samples (blue). A shift in FITC-phalloidin intensity is easily seen in the histogram overlays. The first three histograms show FITC-phalloidin expression on gated lymphocytes, monocytes or neutrophil granulocytes for patient with mutation in ARPC1B. The second and the third row of histograms shows examples of carrier and healthy control.

**Figure 3:**
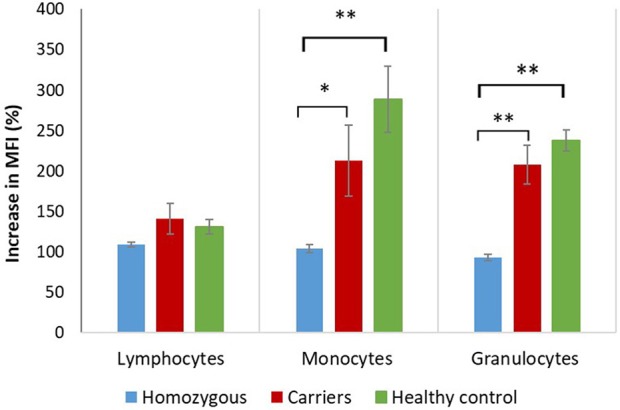
Actin polymerization in patients, carriers and healthy controls. Graphical presentation of mean increase in mean fluorescent intensity (MFI) ± standard error of the mean (SEM) on lymphocytes, monocytes and neutrophil granulocytes. The percentages of increase in fluorescent intensity are presented by setting the fluorescence intensity of unstimulated samples to 100% as described by the equation$\frac{stimulated\;sample*100}{unstimulated\;sample}$. Increase in median fluorescent intensity was analyzed with independent two-tailed Student's *t*-test. Significant differences (^*^p ≤ 0.05; ^**^p ≤ 0.01) between homozygous, carriers and healthy controls are shown.

**Figure 4:**
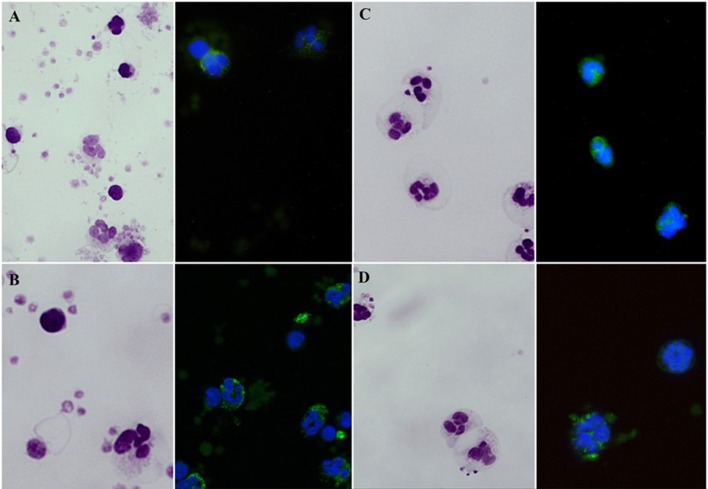
Giemsa stained blood smears and fluorescent microscopy images of FITC-phalloidin stained cells. **(A)** Smears of non-stimulated blood specimen of a healthy subject. **(B)** fMLP-stimulated blood cells of a healthy subject. **(C)** Non-stimulated patient's blood cells. **(D)** fMLP-stimulated patient's blood cells.

Median fluorescence intensities of FITC-phalloidin stained actin in monocytes, lymphocytes, and neutrophils were measured by flow cytometry. The data was normalized to percentage of increase in MFI, to compensate for variation in fluorescence intensity between different days. This calculation is described in the methods. The increase of MFI in FITC-phalloidin stained actin was measured on leukocytes populations from patients, carriers, and healthy controls (Figure 2). In monocytes, increase in MFI in patients was significantly lower than in carriers (*p* = 0.02) or healthy subjects (*p* ≤ 0.01). In lymphocytes, increase in MFI in patients was not statistically significantly lower than in heterozygotic carries (*p* = 0.101) or healthy controls (*p* = 0.108). The most significant differences were observed in neutrophils. In the group with carriers and healthy subjects without ARPC1B mutation, the MFI showed a statistically significant increase (*p* ≤ 0.01) while in the patient's group there was a significant decrease (*p* ≤ 0.01) (Figure 3). Average MFI with or without fMLP stimulation as well as increases in MFI for all three groups are shown in Table 3. Morphological and fluorescence images of fMLP activated and non-activated patient's and healthy subject's phalloidin-marked cells are depicted in Figure 4.

**Table 3:** Average median fluorescence intensity (MFI) of neutrophils and monocytes before and after 20 s stimulation with fMLP in three different groups–homozygous (patients), carriers, and healthy subjects without mutation in ARPC1B.

	**Average MFI FITC-phalloidin stained actin**		
	**Without stimulation**	**With stimulation**	**Increase in MFI**
**Monocytes**			
Homozygous (*n =* 4)	17,382 ± 2,665	17,334 ± 2,113	104 ± 5%
Carriers (*n =* 6)	22,809 ± 7,655	33784 ± 5,684	213 ± 44%
Without mutation (*n =* 12)	26,710 ± 7,050	58,280 ± 14,269	289 ± 41%
**Neutrophils**			
Homozygous (*n =* 4)	4,791 ± 430	4,497 ± 477	94 ± 4%
Carriers (*n =* 6)	6,570 ± 279	13,405 ± 561	208 ± 24%
Without mutation (*n =* 12)	12,701 ± 3,120	31,975 ± 9,503	238 ± 13%
**Lymphocytes**			
Homozygous (*n =* 4)	5,993 ± 404	6,478 ± 380	109 ± 3%
Carriers (*n =* 6)	7,432 ± 1,858	9,226 ± 1,589	141 ± 19%
Without mutation (*n =* 12)	12,611 ± 2,484	15,290 ± 2,672	131 ± 9%

*The results are presented as the mean with the standard error of the mean (SEM)*.

## Discussion

Phalloidin is widely used in studies of actin filament assembly, including analysis of branch formation by Arp2/3 complex. This cyclic peptide binds and stabilizes actin filaments (18). However, the flow cytometry measurement of phalloidin on monocytes and neutrophils in patients with deficiencies in actin polymerization has not yet been considered. Here we have shown that FITC-phalloidin is simple and rapid functional test that can evaluate the last stage of actin polymerization. We identified a missense mutation in the gene ARPC1B resulting in defective actin polymerization in four patients. ARPC1B is prominently expressed in blood/immune cells and is one of two isoforms Arp2/3, which is required for actin filament branching. Thus, ARPC1B deficiency in humans results in defective Arp2/3 actin filament branching that is associated with multisystem disease including platelet abnormalities, cutaneous vasculitis, eosinophilia and predisposition to inflammatory diseases (19). ARPC1B deficiency has a similar multisystem pathogenesis as a lack of expression of WASp, which is also involved in migration and pseudopod formation (20). Mutations in genes that encode actin regulatory proteins in immune cells, give rise to a distinct subset of PIDs. Defects in actin cytoskeleton affect nearly every stage of the immune response: proliferation of hematopoietic cells in the bone marrow, migration, trans-migration through the endothelium to the sight of infection, dramatic shape change needed to phagocytose invading pathogens, presentation of antigens, and the intimate cellular interactions needed for direct cell to cell signaling (1). Immune cells like many other motile cells make 3D actin-filled pseudopodie and navigate through complex environment at speeds of 20 μm/min (20).

Neutrophils respond to chemotactic stimuli, such as fMLP by increasing the nucleation and polymerization of actin filaments. They respond to a gradient of chemoattractant by extending actin-rich pseudopodia preferentially in the direction of the highest concentration of chemotactic molecules. The response to fMLP occur very rapidly, within 5 to 30 s they begin to extend their surface toward the gradient of chemoattractant (21). A few minutes later, neutrophils develop a polarized shape with formation of contracted tail in the rear and F-actin-rich ruffles at the front. Actin acts as the engine that drives neutrophil motility. The cellular amount of polymerized actin can be determined by assessing the content of fluorescent phalloidin, which binds to F-actin in a 1:1 ratio more tightly than to G-actin and, at saturation, the amount of phalloidin bound is a measure of the amount of F-actin present (22). This can be observed with a fluorescence microscope or measured with a flow cytometer (3). Phagocytic cells have very rapid changes in actin polymerization, which is needed for detection and migration toward pathogens, and destroy their targets. According to our results, they are the most appropriate to measure fluorescence intensity of intracellular actin stained with FITC-phalloidin in patients with defect of actin polymerization (Figure 3). Significant difference in fluorescence intensity between patients with mutation in ARPC1B, carriers and healthy controls indicates that the content of polymerized actin in activated monocytes is indeed lower in patients. The same holds for neutrophils, where the observed decrease in actin content below basal levels can be attributed to fMLP-activation induced damage of cells Table 3.

In ARPC1B homozygous patients, we observed increased respiratory burst in neutrophils after mild stimulation with fMLP. Three of our patients with ARPC1B mutation had developed problems with recurrent infection. This could have an effect on the increased oxidative burst after fMLP stimulation, which was not observed in normal blood neutrophils (Figure 1B). It is known that priming of fMLP receptor with cytokines (e.g., TNF-α) facilitates stronger oxidative burst, which in turn has detrimental effect on these oxidizing granulocytes (23, 24). Since higher than normal values of oxidizing granulocytes were also found in PHAGOBURST^TM^ test (Figure 1), we believe the paradoxical decrease in actin content in neutrophils after stimulation can be explained by the mechanism mentioned above. Therefore, activation of cytokine-primed fMLP receptors triggers prominent oxidative burst in granulocytes. Oxidizing cells are consequently damaged in this process, which leads to the release of actin, resulting in lower fluorescence intensity detection (25). The neutrophil and macrophage abnormalities caused by defective actin polymerization might explain increase frequency of bacterial infections.

WASp and Arp2/3 function has been reported to have crucial role in the formation of immunological synapse between dendritic cells (DC) and lymphocytes (26). In WAS patient's, for example, DC uptake of soluble antigen is normal, but phagocytosis and presentation of particulate antigens is impaired (27). Bouma et al. hypothesized that reduced proliferation of lymphocytes is due to impaired antigen presentation and reduced IL-12 release from the DC (28). On the other hand, poor DC migration after antigen uptake may lead to maturation of DCs before they reach lymph nodes, with ectopic cytokine and chemokine release likely to recruit other immune cells that may contribute to inflammatory processes such as eczema (1). All our patients had eczema and all but the younger one had elevated levels of total IgE which is characteristic for WAS and ARPC1B immunodeficiency. However, the youngest patient had increased levels of food-specific IgE. All patients had thrombocytopenia and developed autoimmune diseases.

On lymphocytes, we did not observe significant differences in actin polymerization between homozygotes, asymptomatic heterozygote and healthy controls. The actin polymerization is not as extensive in lymphocytes compare to more motile monocytes and neutrophils. In our group of patients, we have observed mild cellular deficiency (Table 2), which coincides with previous reports (12). A major defect in T cells with abnormal actin polymerization is their altered immunological synapses formation and reduced chemotaxis. This can lead to defective response to CD3 and antigens in some cases (14). However, *in vitro* T-cell proliferation in response to combination of anti-CD3 and anti-CD28, cytokines (IL-15, IL-2) and mitogens was in largely normal (12). In our patients, the response to anti-CD3 and anti-CD28 was normal, but response to mitogen phytohaemagglutinin (PHA) was reduced in two related patients. T lymphocytes successfully participated in immunoglobulin isotype switching, because all patients had increased IgG and IgA levels during the course of their disease. Until now, 14 patients with ARPC1B deficiency have been reported in addition to our four patients, and they all had increased–immunoglobulin E (12, 14, 19).

In summary, by measuring actin polymerization on neutrophils and monocytes with a functional fMLP/phalloidin test, we have efficiently distinguished between symptomatic homozygous patients with mutation in ARPC1B, asymptomatic heterozygous carriers and healthy controls. The mutation of Arp2/3 activator subunit (ARPC1B) resulted in defective polymerization of actin. In blood leukocytes, we have observed the biggest differences in increase of median fluorescence intensity on neutrophils and monocytes. Flow cytometry assays may represent a very useful and rapid tool to detect mutation in ARPC1B that leads to impaired actin polymerization. In the future, it would be very interesting to see if similar differences can be observed also in other primary immunodeficiencies due to abnormalities of actin cytoskeleton, for example Wiskott–Aldrich syndrome.

## Data Availability

The datasets for this manuscript are not publicly available because the data contains some of the personal information from patients. Requests to access the datasets should be directed to AK, andreja-natasa.kopitar@mf.uni-lj.si, MD, marusa.debeljak@kclj.si.

## Ethics Statement

This study was carried out in accordance with the recommendations of the National Ethics Committee and Pediatric committee. The protocol was approved by the Pediatric committee. All subjects gave written informed consent in accordance with the Declaration of Helsinki.

## Author Contributions

AK, MO, and MD performed the experiments and analyzed the data. AK, AI, and MD wrote the paper. All of the authors revised the manuscript, approved the final version submitted for publication and contributed to the conception and design of the study.

### Conflict of Interest Statement

The authors declare that the research was conducted in the absence of any commercial or financial relationships that could be construed as a potential conflict of interest.
